# Dietary oregano aqueous extract improves growth performance and intestinal health of broilers through modulating gut microbial compositions

**DOI:** 10.1186/s40104-023-00857-w

**Published:** 2023-09-01

**Authors:** Fan Zhang, Jiantao Yang, Qinyi Zhan, Hao Shi, Yanhe Li, Dinggang Li, Yingge Li, Xiaojun Yang

**Affiliations:** 1 College of Animal Science and Technology, Northwest A&F University, Yangling, Shaanxi China; 2 Baoding Jizhong Pharmaceutical Corporation, LTD, Baoding, Hebei China; 3 Shaanxi Province Animal Husbandry Technology Extension Station, Xi’an, Shaanxi China

**Keywords:** Broilers, Gut microbiota, Intestinal health, Oregano aqueous extract

## Abstract

**Background:**

Intestinal health plays a pivotal role in broiler chicken growth. Oregano aqueous extract (OAE) effectively exerts anti-inflammatory and antibacterial effects. However, the protective effects of OAE on intestinal health in broilers and the underlying mechanism remain unclear. This study aimed to investigate the potential effects of OAE on growth performance, the gut microbiota and intestinal health. A total of 840 1-d-old male and female broilers (Arbor Acres) were randomly allocated into 6 groups as follows: basal diet (Con), Con + antibiotics (Anti, colistin sulfate 7 g/kg, roxarsone 35 g/kg), Con + 400, 500, 600 and 700 mg/kg OAE (OAE400, OAE500, OAE600 and OAE700). Subsequently, fermentation in vitro together with oral administration trials were carried out to further assess the function of OAE on intestinal health of broilers.

**Results:**

Dietary 700 mg/kg OAE supplementation resulted in an increase (*P* < 0.05) in body weight and a decrease (*P* < 0.05) in feed conversion ratio when compared with the control during d 22 to 42 of the trial. OAE addition resulted in lower (*P* < 0.05) jejunal crypt depth and mRNA expression of *IL-4* and *IL-10* at d 42. In addition, dietary OAE addition increased the abundance of Firmicutes (*P* = 0.087) and *Lactobacillus* (*P* < 0.05) in the cecum, and increased (*P* < 0.05) the content of acetic acid and butyric acid. In the in vitro fermentation test, OAE significantly increased (*P* < 0.05) the abundance of *Lactobacillus*, decreased (*P* < 0.05) the abundance of *unspecified_Enterobacteriaceae*, and increased the content of acetic acid (*P* < 0.05). In the oral administration trial, higher (*P* < 0.05) *IL-4* expression was found in broilers when oral inoculation with oregano fermentation microorganisms at d 42. And SIgA content in the ileum was significantly increased (*P* = 0.073) when giving OAE fermentation supernatant.

**Conclusions:**

Dietary OAE addition could maintain intestinal health and improve growth performance through enhancing intestinal mucosal immunity and barrier function mediated by gut microbiota changes.

**Supplementary Information:**

The online version contains supplementary material available at 10.1186/s40104-023-00857-w.

## Introduction

Intestinal health is closely related to growth performance of animals which depends on light and strong digestion and absorption, complete physical barrier, specific chemical barrier, moderate mucosal immunity and stable microorganism [1]. The gut microbiota also develops most functions including boosting the immune system, improving digestion, and affecting the nervous system [2–4]. Hence, gut microbiota plays a pivotal role in maintaining normal intestinal physiology and health. Necrotizing enteritis caused by excessive proliferation of *Clostridium perfringens* is common in the poultry industry, which leads to huge economic losses. Dietary additives supplementation could strengthen the gut microbiota, intestinal barrier and colonization resistance to pathogens for improving intestinal health.

Natural plants could produce active secondary metabolites and have emerged as safe, easily accessible, and inexpensive sources of feed additives. Oregano has anti-inflammatory, antibacterial and antioxidant effects because it contains terpenoids, phenols and other active substances. A large number of studies have shown that the phenolic hydroxyl in phenolic compounds, as a cationic trans-membrane carrier, caused proton influx and potassium ion outflow, which leads to the loss of proton kinetic force and the obstruction of ATP synthesis, and ultimately damaged bacterial cells [5, 6]. Oregano’s main components is carvacrol and thymol. The phenolic hydroxyl groups contained in carvacrol and thymol can act as hydrogen donors to bind to peroxyradicals in the first step of the oxidation reaction, thus preventing and delaying lipid oxidation [7]. Carvacrol and thymol can enhance the cellular and humoral immunity of broilers [8]. In addition, oregano could greatly improve production performance [9], gut microbiota and microbiota-driven SCFAs [10], and could activate immune responses [11]. However, the underlying mechanism by which oregano improves performance still remain unclear, and the effects of OAE on the intestinal health of broilers await further studies.

Therefore, based on the protective effects of oregano, the present study was performed to evaluate the modulation effect of OAE on growth performance and intestinal health, and reveal the regulation mechanism via fermentation in vitro together with oral administration trials in vivo.

## Materials and methods

### Experimental design, animals, and diets

All animal protocols for this study were approved by the Institutional Animal Care and Use Committee of Northwest A&F University. All broilers used in the study were obtained from the Xianyang Dacheng Poultry Industry Co., Ltd. (Xianyang, China), and housed in three-tier battery cages (cage size: 45 cm × 45 cm × 45 cm). The broiler house was initially set at 35 ℃, but the temperature was gradually decreased to 27 ℃ at week 3 and maintained thereafter. The light cycle was one-hour of darkness per day.

#### Exp. 1

A total of 840 1-d-old male and female broilers (Arbor Acres) were individually weighed and randomly divided into 6 groups with 7 replicates of 20 birds each. Detailed groups are as follows: basal diet without (Con) or with antibiotics (Anti, colistin sulfate 7 g/kg, roxarsone 35 g/kg) or 400, 500, 600 and 700 mg/kg OAE (OAE400, OAE500, OAE600 and OAE700). The study lasted for 42 d. The addition range of OAE was determined as 400–700 mg/kg in our pre-experiment. OAE (carvacrol > 100 g/kg, powder form) was provided by Baoding Jizhong Pharmaceutical Co., Ltd. The powder ingredients were first added to the premixed feed using a step-by-step dilution method, and then mixed with other feed ingredients. The basal diets did not include antibiotics or anticoccidials but included nonstarch polysaccharide degrading enzyme and phytase enzyme. All treatment diets were pelleted after mixing with a conditioning temperature range from 78 to 80 ℃. Each fed a starter diet from 1 to 21 d and a finisher diet from 22 to 42 d (Table1). At d 21 and 42, samples were collected after slaughter.

**Table 1:** Ingredients and nutrients composition of Exp. 1 diets

**Ingredients, %**	**Starter**	**Finisher**
Corn	54.99	67.64
Soybean meal	28.16	17.40
Corn DDGS	8.00	-
Cottonseed meal	4.00	5.00
Corn gluten meal	-	5.00
Soybean oil	0.50	1.17
Mountain flour	1.57	1.24
Calcium hydrophosphate	1.27	1.26
*L*-lysine hydrochloride	0.36	0.45
*DL*-methionine	0.27	0.16
NaCl	0.54	0.26
Mineral premix^a^	0.15	0.15
*L*-threonine	0.09	0.07
Choline chloride	0.08	0.08
Vitamin premix^b^	0.02	0.02
*L*-tryptophan	-	0.11
Phytase	0.02	0.02
Total	100.00	100.00
**Nutritional level**^**c**^, **%**		
Apparent metabolizable energy, kcal/kg	2,673.00	2,922.00
Dry matter	86.41	86.27
Crude protein	21.00	18.50
Crude fat	3.24	3.84
Crude ash	6.26	5.10
Calcium	1.00	0.85
Total phosphorus	0.65	0.58
Available phosphorus	0.34	0.29
Lysine	1.20	1.04

^a^Mineral premix provided the following per kg of the diet: Mn, 95.4 mg; I, 0.38 mg; Fe, 66 mg; Cu, 15 mg; Zn, 96.6 mg; Se, 0.41 mg

^b^Vitamin premix provided the following per kg of the diet: vitamin A, 9,200 IU; vitamin D, 3,000 IU; vitamin E, 38 mg; vitamin K_3_, 3 mg; vitamin B_1_, 3 mg; vitamin B_2_, 10 mg; vitamin B_6_, 5 mg; vitamin B_12_, 0.04 mg; niacin, 40 mg; *D*-calcium pantothenate, 16 mg; folic acid, 2 mg; biotin, 0.3 mg

^c^The nutrient levels were calculated values

#### Exp. 2

Fresh cecal contents were obtained from 42-d-old Arbor Acres broilers fed a basal diet and immediately transferred into an anaerobic chamber. Three volumes of basic culture medium were added to the samples and vortexed until dispersed, then the supernatant was collected. The basic culture medium was prepared according to the method of Chen et al. [12]. Fecal inocula (1%) and without (C group) or with (T group) 1% (w/v) of OAE were mixed with the culture medium, the mixtures were incubated at 37 °C, and culture samples were collected and centrifuged at 12, 24, and 48 h. The microorganism (sediment) of T48 samples (fermented for 48 h in T group) was stored in glycerol, and the supernatant was stored at −80 ℃ for subsequent oral administration trials.

In the oral administration trial, a total of 90 1-d-old male and female broilers (Arbor Acres) were randomized to three groups (six replicates with 5 birds per replicate) as follows: C group (water), S group (supernatant) and M group (microorganism). During d 17–20, each chicken in S and M groups was given orally 1 mL of supernatant, and microorganism (sediment) obtained from the fermentation experiment mentioned, and each chicken in C group was given orally 1 mL of drinking water. The basal diets consisted of monensin, mannanase and phytase enzymes. All birds were fed with the same corn-soybean meal basal diet (crumble form), each fed a starter diet from 1 to 21 d and a finisher diet from 22 to 42 d (Table 2). At d 21 and 42, samples were collected after slaughter.

**Table 2:** Ingredients and nutrients composition of Exp. 2 diets

**Ingredients, %**	**Starter**	**Finisher**
Corn	57.27	60.99
Soybean meal	35.20	30.00
Cottonseed meal	2.00	4.00
Soybean oil	2.00	2.00
NaCl	0.36	0.35
Limestone	2.00	1.70
Calcium hydrogen phosphate	0.30	0.30
Choline chloride	0.05	0.05
*L*-lysine hydrochloride	0.14	0.06
Mineral premix^a^	0.30	0.30
Phytase	0.10	0.10
Vitamin premix^b^	0.03	0.03
*DL*-methionine	0.25	0.12
Total	100.00	100.00
**Nutritional level**^**c**^**, %**		
Apparent metabolizable energy, kcal/kg	2,949.26	3,044.86
Crude protein	21.91	19.89
Calcium	0.96	0.91
Total phosphorus	0.61	0.60
Available phosphorus	0.40	0.40
Methionine	0.57	0.45
Methionine + Cysteine	0.90	0.75
Lysine	1.21	1.05

^a^Mineral premix provided the following per kg of the diet: Mn, 80 mg; I, 0.40 mg; Fe, 80 mg; Cu, 10 mg; Zn, 70 mg; Se, 0.30 mg

^b^Vitamin premix provided the following per kg of the diet: vitamin A, 250,000 IU; vitamin D, 50,000 IU; vitamin K_3_, 53 mg; vitamin B_1_, 40 mg; vitamin B_2_, 120 mg; vitamin B_12_, 0.50 mg; vitamin E, 600 IU; biotin, 0.65 mg; folic acid, 25 mg; pantothenic acid, 240 mg; niacin, 1,000 mg

^c^The nutrient levels were calculated values

### Growth performance

Feed intake and body weight (BW) per pen were measured at d 21 and 42 and used to calculate the average daily gain (ADG), average daily feed intake (ADFI) and feed conversion ratio (FCR, FCR = ADFI/ADG).

### Sample collection

Birds were randomly selected from each replicate and slaughtered, then the middle portion of jejunum (defined as the section between duodenum and ileum) and ileum (defined as the section between Meckel’s diverticulum and ileocecal junction) were isolated and approximately 1 cm segments of the midpoints of jejunum and ileum were fixed in 10% neutral-buffered formalin for histological analysis. The jejunum and ileum mucosa were stored at −80 ℃ for mRNA analysis. Cecum digesta of d 42 were stored at −80 ℃ for analysis of microbial composition.

### UPLC-Q/TOF–MS analysis

Preparation of test solution: 20 mg of oregano aqueous extract was accurately weighed and dissolved in 1 mL 60% methanol solution. The mixture was centrifuged at 10,000 r/min for 20 min, and the supernatant was collected.

The samples were separated at 50 ℃ on a Waters ACQUITY^TM^ UPLC system (Waters Corporation, Milford, MA, USA) equipped with an ACQUITY UPLC HSS T3 column (150 mm × 2.1 mm, 1.8 µm). The mobile phase consisted of solvent A (H_2_O containing 0.1% formic acid, v/v) and solvent B (acetonitrile containing 0.1% formic acid, v/v). The gradient program for biosamples included three segments: 5%–40% B from 0 to 32 min, followed by 40%–95% B from 32 to 37 min. The flow rate was 0.3 mL/min, and the temperature was at 50 ℃ throughout the analysis.

Electrospray ion source (ESI), detected in positive and negative ion modes, scanned in primary and multistage modes, with a scanning range of *m/*z 50 to 1000 and a resolution of 30,000. Ion source voltage is 3.5 kV; The capillary heating temperature is 350 ℃. The flow rate of sheath gas is 40 arb. The flow rate of auxiliary gas is 10 arb. The voltage of tube lens is 120 V; the collision energy of collision-induced dissociation is adjusted to 35% of the maximum value.

The test conditions for UPLC-Q/TOF–MS were set according to the procedure described by Zhou et al. [13]. The corresponding compounds were identified according to the ion mass charge ratio of primary and secondary fragments, the cracking laws of these compounds reported in the literature, and the search and screening of the UNIFI online software.

### Intestinal morphological analysis (Exp. 1)

Jejunal and ileal tissues fixed in formalin were embedded in paraffin, and paraffin sections were sliced using a microtome (Leica Microsystems K. K., Tokyo, Japan) and mounted on glass slides. The sections were dewaxed with xylene, hydrated, and then stained with hematoxylin and eosin (H and E). For each sample, five intact villi-crypt units were selected for morphology observation using a light microscope (Olympus Corporation, Tokyo, Japan) coupled with image processing software (Image J 1.53). Villus height (VH, the height from the tip of the villus to the villus-crypt junction) and crypt depth (CD, the depth of invagination between adjacent villi) were measured. VH to CD ratio (VH/CD) was calculated.

### The concentration of SIgA (Exp. 1 and 2)

The secreted immunoglobulin A (SIgA) content in jejunum and ileum was measured by immunohistochemical staining as described by Wang et al. [14]. The ileum tissue was dewaxed, rehydrated, microwave irradiated, and treated with 3% H_2_O_2_ at room temperature for 25 min, blocked with normal rabbit serum, incubated with the primary antibody overnight at 4℃ (dilution ratio 1:200), incubated with the secondary antibody at room temperature for 50 min (dilution ratio 1:200), and stained by 3,3-diaminoben-zidine (DAB). Finally, the slides were observed with a light microscope (Leica Microsystems K. K., Tokyo, Japan) and Quantitative analysis with Image-Pro Plus software, Version 6.0.

### RNA isolation and real-time quantitative PCR (Exp. 1 and 2)

Total RNA was extracted from the jejunum and ileum mucosa following Trizol Reagent protocol (AG21102, Accurate Biotechnology (Hunan) Co., Ltd., Changsha, China). The purity and concentration of the total RNA were measured and cDNA was synthesized with an Evo M-MLV RT Kit for qPCR (AG11707, Accurate Biotechnology (Hunan) Co., Ltd.). The mRNA expression was analyzed with a SYBR® Green Premix Pro Taq HS qPCR Kit (AG11701, Accurate Biotechnology (Hunan) Co., Ltd.) on the iCycler IQ5 (Bio-Rad, Hercules, CA, USA). Primer sequences used in this study are shown in Table 3. The reaction conditions were as follows: 95 ℃ for 30 s; 40 cycles of 95 ℃ for 5 s, 60 ℃ for 30 s. Each sample was measured in duplicate and the relative mRNA expression levels were analyzed using β-actin as an internal control by the 2^−ΔΔCt^ method.

**Table 3:** Sequences of real-time PCR primers

Genes	Primer sequence (5’ → 3’)	Accession No.
*IL-4*	F: AGCACTGCCACAAGAACC	NM_001398460.1
	R: GCTAGTTGGTGGAAGAAGGTA	
*IL-10*	F: CGCTGTCACCGCTTCTTCA	NM_001004414.3
	R: CGTCTCCTTGATCTGCTTGATG	
*TNF-α*	F: TATGTGCAGCAACCCGTAGT	NM_204267.2
	R: AACAACCAGCTATGCACCCCA	
*MUC2*	F: AGCGAGATGTTGGCGATGAT	NM_001318434.1
	R: AAGTTGCCACACAGACCACA	
β-actin	F: ATTGTCCACGCAAATGCTTC	L08165
	R: AAATAAAGCCATGCCAACTCGTC	

*F* Forward primer, *R* Reverse primer, *IL* Interleukin, *TNF-α* Tumor necrosis factor-α, *MUC2* Mucin 2

### Microbiota analysis (Exp. 1 and 2)

The microbiota analysis was commissioned by Microeco Tech Co., Ltd. (Shenzhen, China), and the methods were performed according to the procedure described by Peng et al. [15]. The V3-V4 hypervariable regions of the bacteria 16S rRNA gene were amplified with primers 338F (5’-ACTCCTACGGGAGGCAGCAG-3’) and 806R (5’-GGACTACHVGGGTWTCTAAT-3’) by thermocycler PCR system (GeneAmp 9700, ABI, USA). Purified amplicons were pooled in equimolar and paired-end sequenced (2 × 300) on an Illumina MiSeq platform (Illumina, San Diego, USA) according to the standard protocols. The analysis was conducted by following the “Atacama soil microbiome tutorial” of QIIME2docs along with customized program scripts (https://docs.qiime2.org/2019.1/). Briefly, raw data FASTQ files were imported into the format which could be operated by QIIME2 system using qiime tools import program. Demultiplexed sequences from each sample were quality filtered and trimmed, de-noised, merged, and then the chimeric sequences were identified and removed using the QIIME2 DADA2 plugin to obtain the feature table of amplicon sequence variant (ASV). The QIIME2 feature-classifier plugin was then used to align ASV sequences to a pre-trained GREENGENES 13_8 99% database (trimmed to the V3V4 region bound by the 338F/806R primer pair) to generate the taxonomy table. Any contaminating mitochondrial and chloroplast sequences were filtered using the QIIME2 feature-table plugin. Feature level alpha diversity indices, such as observed OTUs, Chao1 richness estimator, Shannon diversity index, and Faith’s phylogenetics diversity index were calculated to estimate the microbial diversity within an individual sample. Beta diversity distance measurements, including Bray-Curtis, unweighted UniFrac and weighted UniFrac were performed to investigate the structural variation of microbial communities across samples and then visualized via principal coordinate analysis (PCoA) and nonmetric multidimensional scaling (NMDS) [16].

### Measurement of short-chain fatty acids (Exp. 1 and 2)

Approximately 0.3 g of fecal samples were thawed and diluted with 1 mL of ultrapure water, and 0.5 mL supernatant was obtained by centrifuging at 13,500 r/min for 10 min. Then the supernatant was mixed with 0.1 mL of 25% metaphosphoric acid solution and the mixed solution was placed at 4 ℃ for 4 h before centrifuging at 13,500 r/min for 15 min, afterwards the 0.4 mL of supernatant was mixed with 0.1 mL of 25% crotonic acid and the mixed solution was placed at 4 ℃ for 1 h, filtered by 0.45 μm filter (Millipore Co., Bedford, MA, USA). SCFAs concentrations were determined via gas chromatography (Agilent 7890A, Agilent Technologies, Santa Clara, CA, USA) according to the procedures described by Shen et al. [17], the chromatography was performed on Shodex RSpak KC-811 column (6 μm, 8.0 mm × 300 mm). The formula for calculating the concentration of short-chain fatty acids is as follows: 

$${\mathrm C}_{(\mathrm{SCFAs})}={\mathrm S}_{(\mathrm{SCFAs})}\times{\mathrm C}_{(\mathrm{crotonic}\;\mathrm{acid})}\times\mathrm k/{\mathrm S}_{(\mathrm{crotonic}\;\mathrm{acid})},\mathrm k={\mathrm C}_{(\mathrm{standard}\;\mathrm{substances})}/{\mathrm C}_{(\mathrm{crotonic}\;\mathrm{acid})}$$

C is the concentration of SCFAs, S is the area of the corresponding peak, and k is the concentration of the corresponding SCFA standard substances/the concentration of crotonic acid.

### Statistical analysis

Data were expressed as means with standard error of mean (SEM). The data were analyzed for the homogeneity of variances and normality using Levene’s and Shapiro–Wilk’s tests, respectively. C group and T group in Exp. 2, Student’s *t*-test was applied to the normal data, the heterogeneous or non-normally-distributed data were analyzed using Mann–Whitney U-test, and pairwise differences in rank sums were evaluated using selected comparisons tests. For all other variables in Exp. 1 and C, M and T group in Exp. 2, the normal data were assessed for statistical significance using a one-way analysis of variance (ANOVA) and Duncan’s multiple range test for pairwise comparisons. The heterogeneous or non-normally-distributed data were analyzed using a non-parametric Kruskal–Wallis test, and pairwise differences in rank sums were evaluated using selected comparisons tests. IBM SPSS 26.0 (Chicago, IL, USA) was used to perform statistical analysis. Statistical significance was considered at *P* < 0.05 and trends at *P* < 0.1.

## Results

### Composition analysis of OAE

Fifteen flavonoids, including seven flavonoid glycosides and eight flavonoid aglycones, were identified from oregano aqueous extract by UPLC-Q-TOF–MS. There were 5 phenylpropanoids and their esters or glycosides. The top ingredients were flavonoids, such as vicenin-2, taxifolin and eriodictyol (Fig. S1).

### OAE improved the performance of broilers (Exp. 1)

As shown in Table 4, dietary 700 mg/kg OAE addition significantly improved (*P* < 0.01) BW at d 42 in comparison with the control. At the same time, the supplementation of 700 mg/kg OAE enhanced (*P* < 0.01) the ADG from 22 to 42 d and from 1 to 42 d. In addition, 700 mg/kg OAE significantly reduced (*P* < 0.05) the FCR from 22 to 42 d, but OAE had no effect on ADFI.

**Table 4:** Effects of dietary supplementation with oregano aqueous extract on growth performance of broilers^1 ^(Exp. 1)

**Items**	**Treatments**^2^						**SEM**^3^	***P-value***
	**Con**	**Anti**	**OAE400**	**OAE500**	**OAE600**	**OAE700**		
d 1–21								
BW, g	1,017.52^ab^	1,066.22^a^	997.60^b^	997.26^b^	1,035.96^ab^	1,043.10^ab^	7.321	0.030
ADG, g	46.55^ab^	48.87^a^	45.60^b^	45.58^b^	47.43^ab^	47.77^ab^	0.349	0.030
ADFI, g	67.61	70.36	67.39	66.80	71.55	70.61	0.578	0.069
FCR	1.48	1.45	1.51	1.48	1.48	1.51	0.011	0.630
d 22–42								
BW, g	2,847.05^c^	3,102.39^b^	3,010.53^bc^	2,940.42^bc^	3,016.13^bc^	3,278.39^a^	31.194	< 0.001
ADG, g	87.12^c^	96.96^b^	85.85^bc^	92.53^bc^	94.29^bc^	106.44^a^	1.417	0.001
ADFI, g	197.28	183.17	194.31	175.48	186.67	202.28	3.583	0.300
FCR	2.28^a^	1.90^b^	2.03^ab^	1.91^b^	1.91^b^	1.91^b^	0.040	0.019
d 1–42								
ADG, g	66.83^c^	72.91^b^	70.73^bc^	69.06^bc^	70.86^bc^	77.10^a^	0.743	< 0.001
ADFI, g	64.63	70.36	66.76	65.50	68.06	67.06	1.020	0.685
FCR	1.86	1.72	1.81	1.75	1.78	1.72	0.028	0.650

^1^ *n* = 7 replicates per treatment

^2^Con, OAE400, OAE500, OAE600 and OAE700, broilers received a basal diet supplemented with 0, 400, 500, 600 or 700 mg/kg oregano aqueous extract, respectively; Anti, broilers received a basal diet supplemented with 7 g/kg mycolistin sulfate and 35 g/kg locke sand arsine

^3^*SEM* Standard error of the mean

^a,b,c^Values within a row with no common superscripts differ significantly (*P* < 0.05)

### OAE affected intestinal health (Exp. 1)

**Fig. 1:**
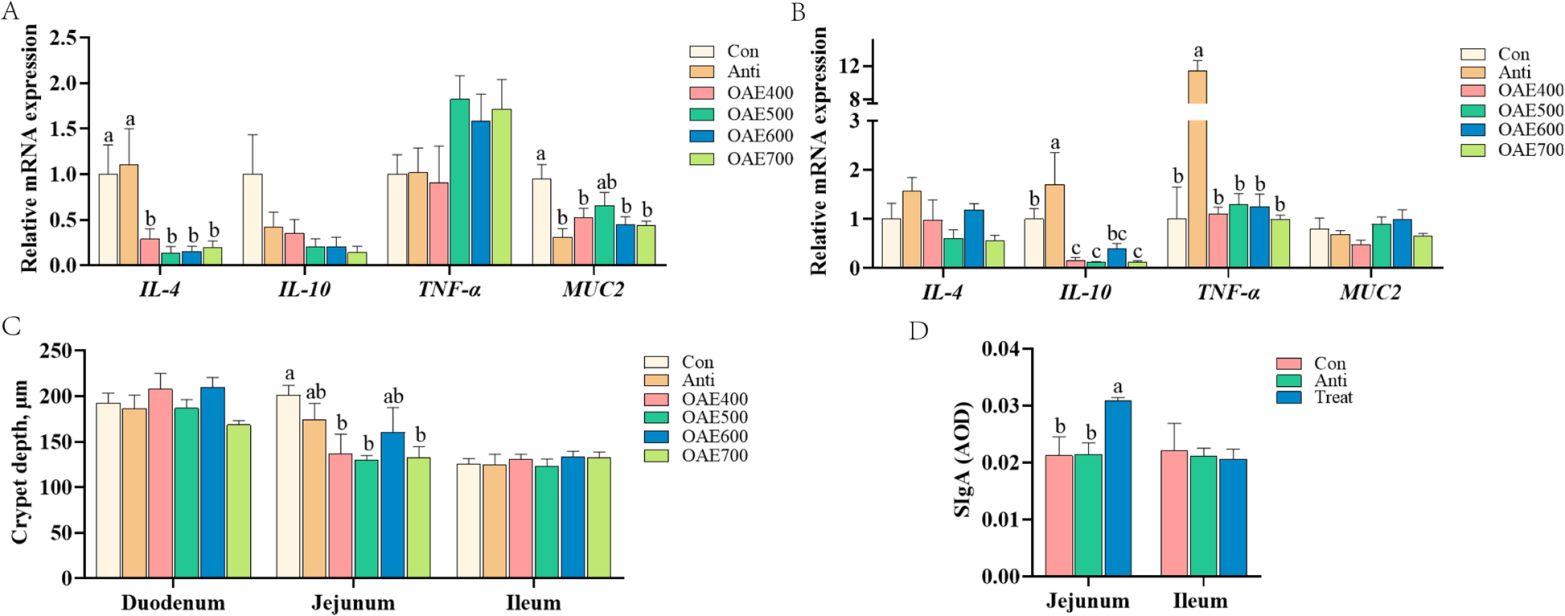
Oregano aqueous extract (OAE) affected intestinal health (Exp. 1). The relative mRNA expression of *IL-4*, *IL-10*, *TNF-α* and *MUC2* in jejunum (**A**) and ileum (**B**) at d 42. Intestinal crypt depth at d 42 (**C**). At d 42, the secretion of SIgA in jejunum and ileum (**D**). Data are expressed as means ± standard deviation. ^a−c^Treatments with no common superscripts differ significantly (*P* < 0.05). *IL*, interleukin; *TNF-α*, tumor necrosis factor-α; *MUC2*, mucin 2; SIgA, Secreted immunoglobulin A

In order to investigate the influence of OAE on intestinal mucosal immunity, the relative mRNA expression of cytokines and the secretion of SIgA in intestinal mucosa were measured. With regard to d 21, no significant effect (*P* > 0.05) was found on interleukin-4 (*IL-4*), interleukin-10 (*IL-10*) and tumor necrosis factor-α (*TNF-α*) expression (Fig. S2A and B). At d 42, the mRNA levels of *IL-4* and *IL-10* in the jejunum decreased significantly (*P* < 0.05) with the OAE in a dose-dependent manner (Fig. 1A), and dietary supplementation with OAE down-regulated (*P* < 0.05) the relative mRNA expression of *IL-10* in the ileum (Fig. 1B). At d 21, 400 mg/kg OAE significantly increased the secretion of mucin 2 (*MUC2*) in the jejunum (Fig. S2A). Nevertheless, jejunal mucin 2 expression was significantly decreased (*P* < 0.05) with OAE addition, except in OAE500 group at d 42 (Fig. 1A). In the jejunum, intestinal crypt depth was significantly decreased (*P* < 0.05) in OAE supplementation group, and OAE supplementation at 500 mg/kg and 700 mg/kg showed the best improvement (Fig. 1C). Based on the above results, OAE700 group was used to carry out subsequent studies, and changed its name to Treat. Results showed that the secretion of SIgA increased significantly (*P* < 0.05) in jejunum at d 42 with OAE addition, but there was no change in the ileum (Fig. 1D).

### OAE modulated gut microbiota and SCFAs (Exp. 1)

**Fig. 2:**
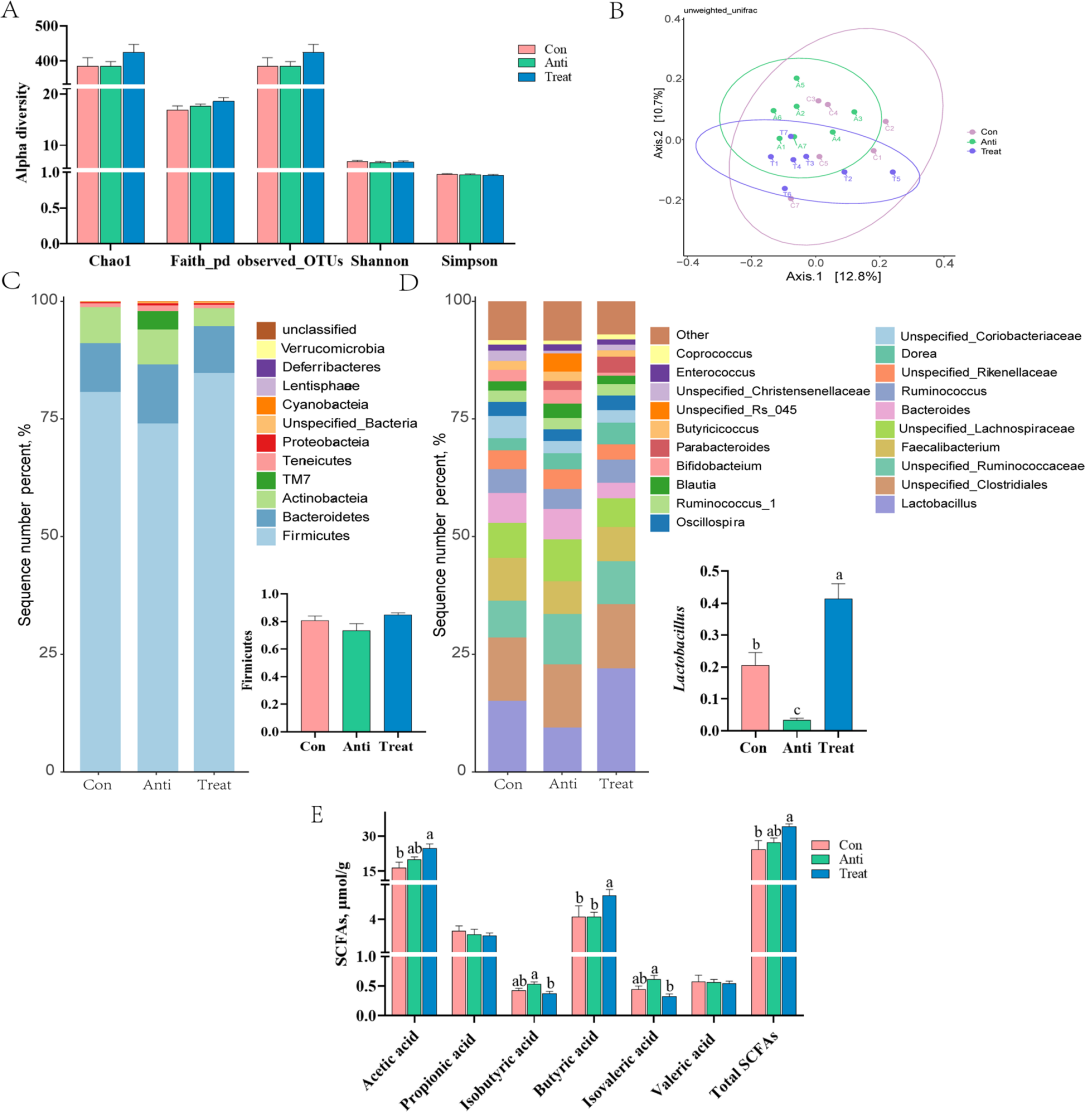
Oregano aqueous extract (OAE) modulated gut microbiota and SCFAs (Exp. 1). Alpha (**A**) and Beta (**B**) diversity analysis of cecum microbiota from broilers. Beta diversity analysis with principal coordinates analysis (PCoA) was based on the unweighted UniFrac distance. Relative abundance of microbiota at the phylum (**C**) and genus (**D**) level. Short-chain fatty acid content of cecal digesta (**E**). Data are expressed as means ± standard deviation. ^a−c^Treatments with no common superscripts differ significantly (*P* < 0.05). PCoA, Principal coordinate analysis; SCFA, Short-chain fatty acid

We further explored the regulation of OAE on gut microbiota. In alpha diversity indexes, there was no influence (*P* > 0.05) on Chao1, Faith_pd, observed_OTUs, Shannon and Simpson indices among all groups (Fig. 2A). Beta diversity analysis was illustrated by principal coordinate analysis (PCoA), the results based on the unweighted UniFrac distance showed separation of microbial communities between Anti and OAE-supplemented groups (*P* < 0.05; Fig. 2B). To further understand the specific changes in the microbial community, we analyzed the microbiota taxonomic composition. At the phylum level, Firmicutes abundance was somewhat reduced in the Anti group, and increased (*P* = 0.087) with the OAE supplementation and returned to normal levels. The ratio of Firmicutes/Bacteroidetes was higher (*P* = 0.067) in OAE than those in Con and Anti group (Fig. 2C). At the genus level, *Lactobacillus* was decreased significantly (*P* < 0.05) in the Anti group and reversed in the OAE group (Fig. 2D). The SCFAs results showed that OAE could increase the contents of acetic acid, butyric acid and total SCFAs (*P* < 0.05), while the contents of isobutyric acid and isovaleric acid was decreased (*P* < 0.05; Fig. 2E).

### Effects of OAE on microorganisms and SCFAs in vitro (Exp. 2)

**Fig. 3:**
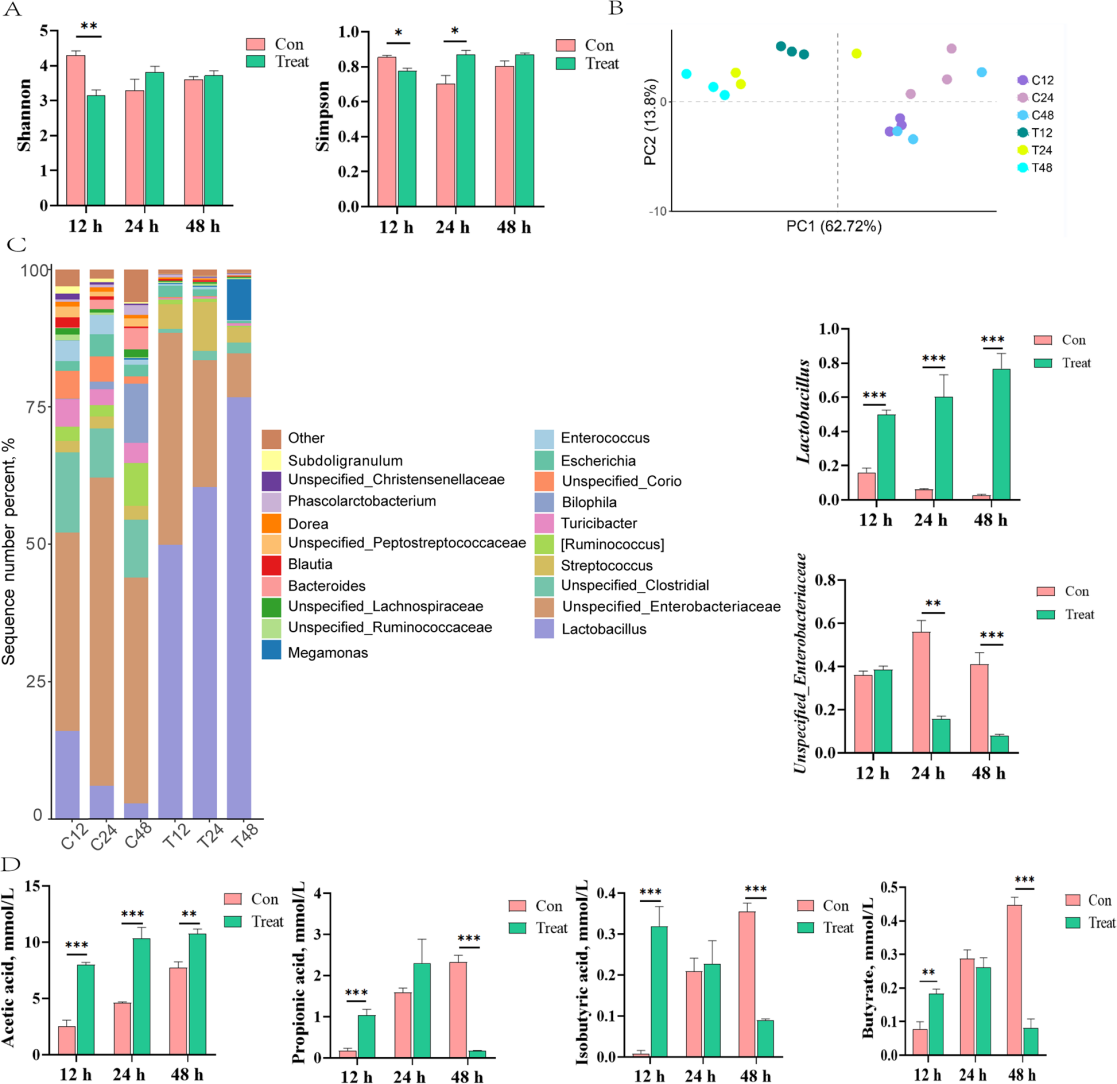
Effects of oregano aqueous extract (OAE) on microorganisms and SCFAs in vitro (Exp. 2). Alpha (**A**) and Beta (**B**) diversity analysis of microorganisms. Beta diversity analysis with principal coordinates analysis (PCoA) was based on the unweighted UniFrac distance. Relative abundance of microbiota at the genus (**C**) level. Content of short-chain fatty acids in fermentation supernatant (**D**). Data are expressed as means ± standard deviation. ^*^P < 0.05, ^**^P < 0.01, ^***^P < 0.001

Then, in order to explore the direct effect of OAE on gut microbiota, OAE was fermented together with cecum microbiota in vitro. The results showed that both Shannon and Simpson indices were decreased in the OAE group at 12 h, while Simpson indices were increased in the presence of OAE at 24 h (*P* < 0.05; Fig. 3A). Additionally, principal components analysis (PCA) result displayed that the microbial community structure in Treat group was significantly different from Con group at each time period, the composition of microorganisms within the groups was relatively similar (Fig. 3B). It is noteworthy that the abundance of *Lactobacillus* in the Treat group was significantly higher (*P* < 0.05) than that in the Con group at each time period. Similar observations can be obtained in previous experiments. However, the abundance of *unspecified_Enterobacteriaceae* in the Treat group decreased significantly (*P* < 0.05) compared with the Con group at 24 h and 48 h (Fig. 3C). In addition, the concentration of acetic acid in the Treat group was significantly higher (*P* < 0.05) than that in the Con group at each time period, while the content of propionic acid and butyric acid in the Treat group was significantly reduced (*P* < 0.05) after 24 h. The content of isobutyric acid was increased in Con group and decreased with time in Treat group (*P* < 0.05; Fig. 3D).

### OAE regulated intestinal health mediated by gut microbiota (Exp. 2)

**Fig. 4:**
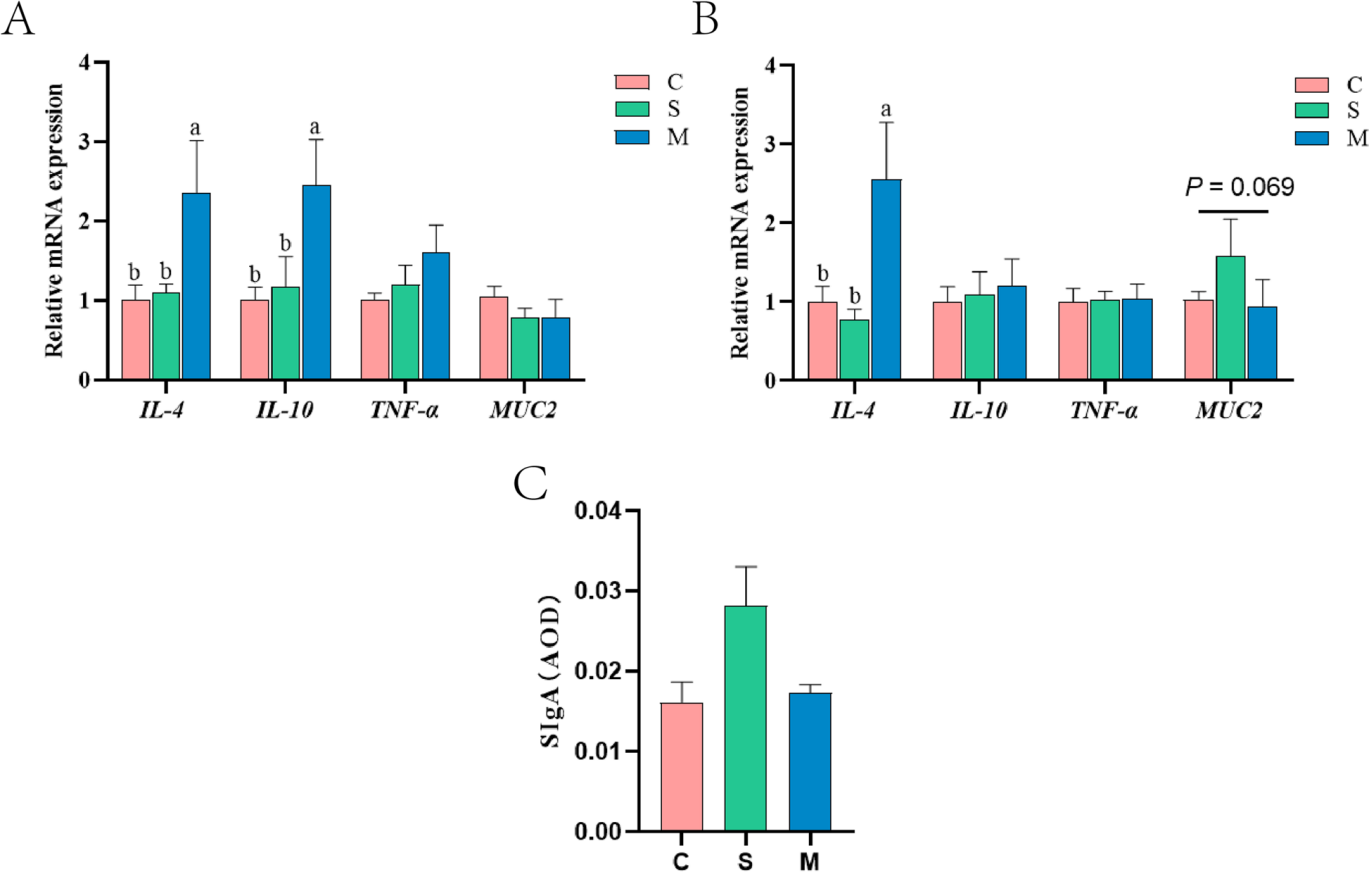
Oregano aqueous extract (OAE) mediated intestinal health by gut microbiota (Exp. 2). The relative mRNA expression of *IL-4*, *IL-10*, *TNF-α* and *MUC2* in jejunum (**A**) and ileum (**B**) at d 42. At d 42, the secretion levels of SIgA in ileum (**C**). Data are expressed as means ± standard deviation. ^a−c^Treatments with no common superscripts differ significantly (*P* < 0.05). *IL*, interleukin; *TNF-α*, tumor necrosis factor-α; *MUC2*, mucin 2; SIgA, Secreted immunoglobulin A

To investigate whether OAE regulated intestinal health through gut microbiota, we gavaged oregano fermentation supernatant or microbe to verify the role of microbe in regulating intestinal health. The results showed that the gavage of microbe significantly increased (*P* < 0.05) the relative mRNA expression of *IL-4* in jejunum at d 21 (Fig. S3A and B). And the relative mRNA expression of *IL-4* was significantly increased in M group in the jejunum and ileum at d 42, and the mRNA expression of *IL-10* was significantly increased through microbial regulation in jejunum at d 42 (*P* < 0.05; Fig. 4A and B). At d 42, the mRNA expression of mucin 2 in the ileum showed an upward trend in S group (*P* = 0.069; Fig. 4B). In addition, gavage of supernatant increased the secretion of SIgA in the ileum (*P* = 0.073; Fig. 4C).

## Discussion

In the present study, dietary OAE supplementation increased body weight, average daily gain and decreased feed conversion ratio in broilers and in a dose-dependent manner. Consistent with our findings, several recent studies have indicated that oregano extract improved the growth performance [18–20]. Aromatic substances of phytogenic feed additives were also reported to stimulate the intestinal secretion of digestive enzymes in broilers, increase absorption area and contribute to the stabilization of the gut microbiota, and growth performance improvement [21]. In addition, in this study, the reduction of intestinal crypt depth, improved immune homeostasis and altered microorganisms structure all suggested the role of OAE in enhancement of digestion absorption function and maintaining healthy intestinal condition as well as benefiting feed utilization in broiler.

Inflammatory reactions result in the production of numerous cytokine and inflammatory mediators, causing tissue and intestinal epithelial cell damage, which decreases intestinal barrier functions [22, 23]. In this study, the mRNA expression levels of *IL-4* and *IL-10* were decreased in the OAE group at d 42. This result might be associated with flavonoids in OAE (including apigenin and toxifolin), which were reported to inhibit Th2-type cytokine production [24–26]. Berry polyphenol components such as flavonoids, proanthocyanidins, and anthocyanins have developed functions in suppressing the secretion of cytokines such as *IL-4* [27]. In addition, the decreased expression of *IL-4* and *IL-10* might be due to the inhibition of intestinal pathogens by OAE and the reduction of the intestinal inflammatory response, so there is no need to secrete excessive anti-inflammatory factors. Furthermore, it has been demonstrated that SIgA acts as the first-line defense barrier in protecting the intestinal epithelium. In the present study, dietary OAE addition significantly increased SIgA secretion [18]. Studies have found that SIgA could enhance the immune function of the intestinal mucosa [28]. SIgA was dysregulated and led to the change of microbial communities [29]. And the relationship between beneficial bacteria and IgA was bilateral, which contribute to improving the intestinal barrier. Thus, the reduced inflammatory response and improved intestinal barrier in response to OAE treatment would be beneficial for the maintenance of intestinal health and growth performance.

To better understand the positive effects of OAE, further analysis was conducted on gut microbiota. There are hundreds of millions of microorganisms in the intestine. The gut microbiota interactions play an important role in preventing pathogen colonization, maintaining immune homeostasis and nutrient metabolism. The active ingredient of oregano (such as thymol and carvacrol) has strong lipid solubility, which can quickly penetrate the cell membrane of pathogenic microorganisms, change their permeability and cause the loss of contents. Moreover, it could effectively prevent the oxidative energy supply process of mitochondria, damaging pathogenic microorganisms due to lack of energy [6, 30]. Apigenin and toxifolin have been reported to significantly increased the abundance of *Lactobacillus* and inhibited the reproduction of *E. coli* [31–33]. In this study, OAE treatment increased the abundance of some beneficial bacteria such as *Lactobacillus*, which were helpful for the maintenance of the overall microbial structure. *Lactobacillus* is recognized as beneficial bacteria which can promote the growth of animals, regulate the normal flora of the gastrointestinal tract and improve the body’s immunity. Intestinal colonization resistance means the inhibition of resident bacteria overgrowth within the intestinal tract. *Lactobacillus* can maintain intestinal colonization resistance and resist invasion [34, 35]. Thus, these results indicated that OAE effectively inhibits the colonization of pathogenic bacteria, which may be related to apigenin and toxifolin. Invasion of pathogens can cause secretion of pro-inflammatory factors such as IL-1β and TNF-α [36], while beneficial bacteria could resist the invasion of pathogens and inhibit the increase of pro-inflammatory factors. As reported, Firmicutes is the dominant species in poultry and most of them are beneficial bacteria. The ratio of Firmicutes/Bacteroidetes was usually used to represent the distribution of beneficial and harmful bacteria. These beneficial bacteria, such as all members of the *Lactobacillus* family, maintained intestinal health by modulating cytokine and chemokine gene expression [37, 38]. In this study, gut microbiota structure alteration might conduce to the enhanced intestinal barrier function and alleviate inflammation, thereby improving intestinal health.

Beneficial bacteria in the gut could ferment carbohydrates to produce short-chain fatty acids, which also inhibit the growth of harmful bacteria [39, 40]. Short-chain fatty acids provide energy for epithelial cell to meet the requirements of epithelial barrier function and cell division. SCFAs are regarded as mediators in the communication between the gut microbiota and the immune system [41–43]. Especially, acetate could regulate intestinal inflammation by stimulation of GPR43 [44], helping to maintain intestinal epithelial barrier function [45]. It was reported that the main components of short-chain fatty acids were acetic acid, propionic acid and butyric acid, accounting for more than 95%, among which acetic acid content was the highest [46], which was consistent with our experimental results. It has been reported that dietary acetic acid could increase appetite and regulate metabolism [47, 48]. Therefore, the increase in growth performance might be positively correlated with acetic acid content. Butyric acid as the main substitute for Firmicutes could also develop function in anti-inflammatory and regulating gut microbiota [49, 50]. In the current study, OAE increased the abundance of *Lactobacillus* and Firmicutes, then increased the contents of acetic acid and butyric acid, thereby maintaining intestinal health.

OAE contains not only alcohol-soluble substances but also water-soluble substances, which contain the complete complex of the plant [51]. Moreover, the contents of total phenols and flavonoids in aqueous extract are high [52]. Quercetin, apigenin and other flavonoids have low bioavailability and need to be metabolized by hindgut microbiota [53, 54]. This suggests that OAE had the potential to improve the growth performance in birds through modulating gut microbiota. Subsequently, cecal microorganisms were added to the basal medium with OAE, and the enriched microorganisms and their metabolites were orally administered to broilers to study the direct effect of microorganisms on intestinal health. The results showed that *Lactobacillus* was increased and *Unclassified_Enterobacter* was decreased in the OAE group. *Enterobacter* is the most common cause of Gram-negative bacterial infection. It mainly includes *Yersinella*, *Escherichia coli*, *Klebsiella* and so on. *E. coli* produced toxins that disrupted the intestinal barrier, causing disorders of the gut microbiota and metabolic diseases [55]. Gut homeostasis is mediated by the preponderance of obligate anaerobic members of Firmicutes and Bifidobacteriaceae, whereas the increase in facultative anaerobic Enterobacteriaceae is a common marker of gut dysbiosis [56]. However, the gut microbiota imbalance appeared in the absence of OAE. These results indicated that the OAE can directly affect the microorganisms, and has good antibacterial activity in vitro, which was consistent with the results in vivo in the study. In addition, OAE increased SCFAs contents, then propionic and butyric acids might be converted to acetic acid in the absence of carbon sources, resulting in a decrease in their concentrations after 24 h.

Subsequently, we evaluated the direct regulation of the gut microbiota and the role of SCFAs driven by the microbiota on intestinal health. The results showed that the mRNA expression levels of *IL-4* and *IL-10* increased in microorganism group to a certain extent, while the mRNA expression levels of *MUC2* and secretion of SIgA were increased in supernatant group. *Lactobacillus plantarum* increased the mRNA expression of *IFN-γ* and *IL-4* in the jejunum of broilers [57, 58]. The results implied that enriched *Lactobacillus* could regulate the expression of cytokines to improve intestinal health. In addition, it was reported that acetic acid could maintain the integrity of intestinal epithelium [59]. Burger-van Paassen et al. [60] found that acetic acid, propionic acid and butyric acid could all improve the expression of *MUC2* gene and protein in LS174T cells. SCFAs were involved in the activation of B cells, thereby promoting the secretion of SIgA. The excellent effects in supernatant group may be mainly caused by acetic acid. Therefore, OAE could promote the growth of *Lactobacillus*, and inhibit the growth of harmful bacteria, consequently improving mucosal immunity. While the special microbiota could drive the production of acetic acid, which could enhance the protection of mucin and SIgA in the intestine.

## Conclusions

**Fig. 5:**
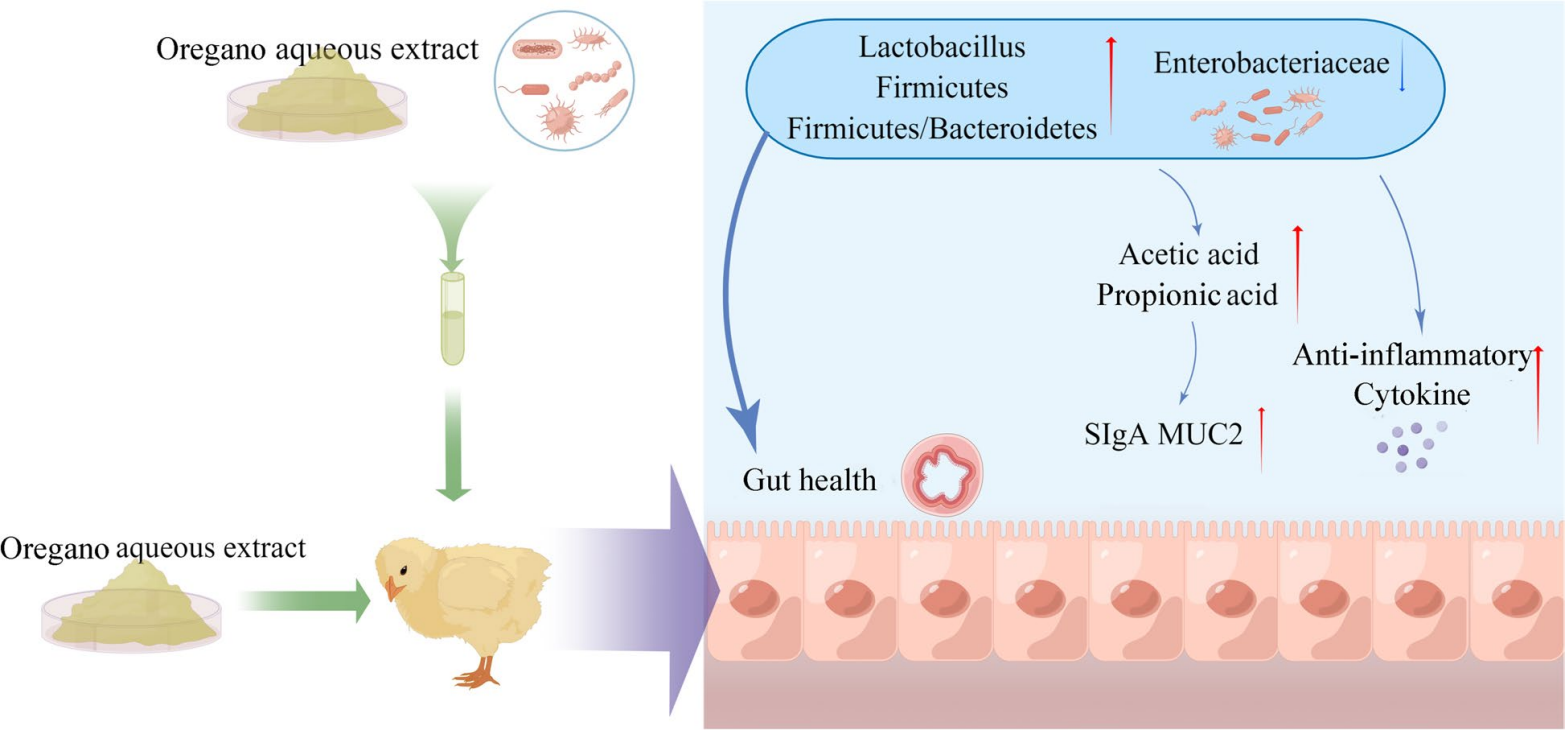
Proposed functions of oregano aqueous extract (OAE) in broilers. Items with a red up-arrow indicated the increased bacteria, SCFAs, SIgA or mucosal gene expression in the OAE-supplemented group compared to the control, whereas those with a blue down-arrow indicated the decreased ones in the OAE-supplemented group

In conclusion, our results demonstrated a potential beneficial role of OAE in improving the growth performance and intestinal health in broilers, which may be related to the improvement of intestinal barrier function and mucosal immunity mediated by microbial changes (Fig. 5). These findings support the potential application of OAE as a safe and effective nutritional intervention strategy to maintain intestinal health and enhance growth performance in broilers.

## Supplementary Information

**Additional file 1: Fig. S1.** Identification of OAE. **Fig. S2.** OAE affected intestinal morphology and mucosal immunity. The relative mRNA expression of *IL-4*, *IL-10*, *TNF-α* and *MUC2* in jejunum (**A**) and ileum (**B**) at d 21. Intestinal villus height (**C**) and villus height/crypt depth (**D**) at d 42. Data are expressed as means ± standard deviation. ^a−c^Treatments with no common superscripts differ significantly (*P* < 0.05). *IL*, interleukin; *TNF-α*, tumor necrosis factor-α; *MUC2*, mucin 2. **Fig. S3.** Microorganism and supernatant for intestinal health. The relative mRNA expression of *IL-4*, *IL-10*, *TNF-α* and *MUC2* in jejunum (**A**) and ileum (**B**) at d 21. At d 42, the secretion levels of SIgA in jejunum (**C**). Data are expressed as means ± standard deviation. ^a−c^Treatments with no common superscripts differ significantly (*P* < 0.05). *IL*, interleukin; *TNF-α*, tumor necrosis factor-α; *MUC2*, mucin 2; SIgA, Secreted immunoglobulin A.

## Data Availability

The data produced or analyzed during the current study are available from the corresponding author by reasonable request.

## Abbreviations

ADFI: Average daily feed intake
ADG: Average daily gain
BW: Body weight
FCR: Feed conversion ratio
IL: Interleukin
MUC2: Mucin 2
OAE: Oregano aqueous extract
PCA: Principal components analysis
PCoA: Principal coordinate analysis
SCFA: Short-chain fatty acid
SIgA: Secreted immunoglobulin A
TNF-α: Tumor necrosis factor-α
