# Supplementary material for: Dietary oregano aqueous extract improves growth performance and intestinal health of broilers through modulating gut microbial compositions
Source: J Anim Sci Biotechnol. 2023 Sep 1;14:77. doi: 10.1186/s40104-023-00857-w (PMC10472629; doi:10.1186/s40104-023-00857-w)
Supplement: Supplementary file 1 — Additional file 1: Fig. S1. Identification of OAE. Fig. S2. OAE affected intestinal morphology and mucosal immunity. The relative mRNA expression of IL-4, IL-10, TNF-α and MUC2 in jejunum (A) and ileum (B) at d 21. Intestinal villus height (C) and villus height/crypt depth (D) at d 42. Data are expressed as means ± standard deviation. a−cTreatments with no common superscripts differ significantly (P < 0.05). IL, interleukin; TNF-α, tumor necrosis factor-α; MUC2, mucin 2. Fig. S3. Microorganism and supernatant for intestinal health. The relative mRNA expression of IL-4, IL-10, TNF-α and MUC2 in jejunum (A) and ileum (B) at d 21. At d 42, the secretion levels of SIgA in jejunum (C). Data are expressed as means ± standard deviation. a−cTreatments with no common superscripts differ significantly (P < 0.05). IL, interleukin; TNF-α, tumor necrosis factor-α; MUC2, mucin 2; SIgA, Secreted immunoglobulin A. [file 40104_2023_857_MOESM1_ESM.docx]

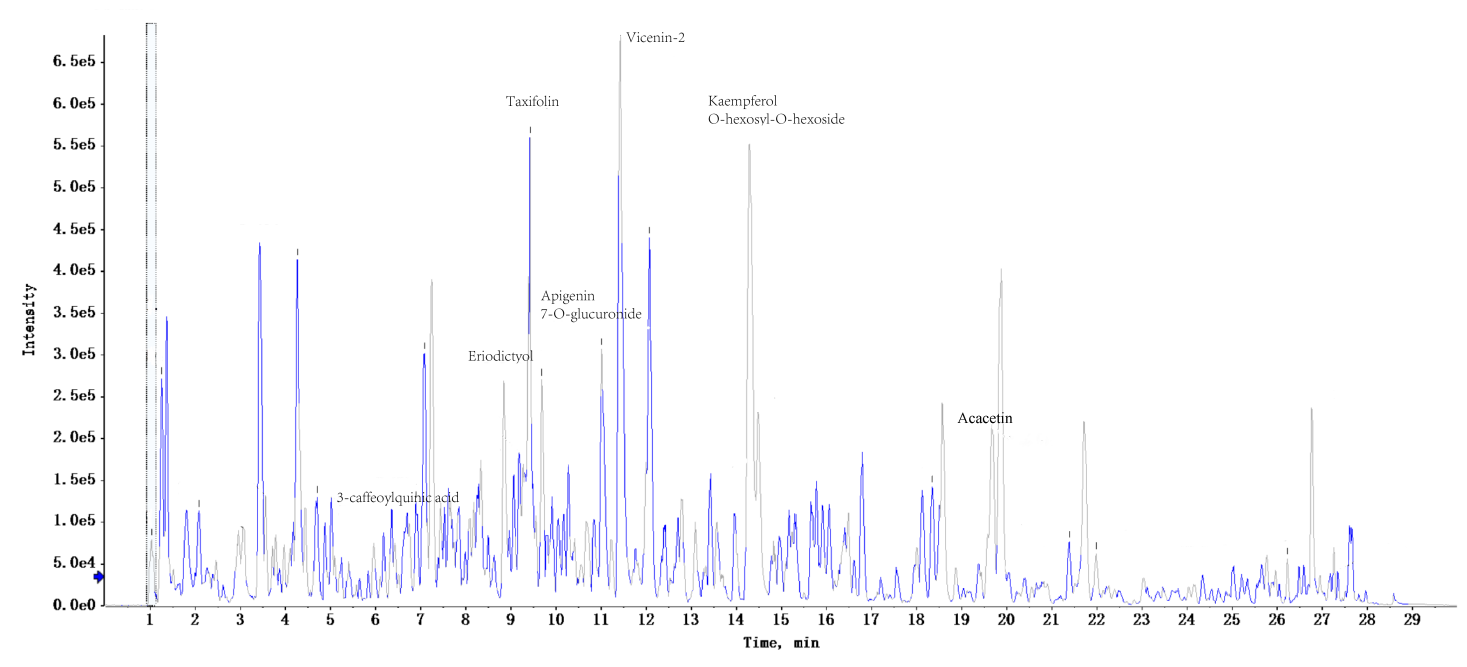


**Fig. S1** Identification of OAE


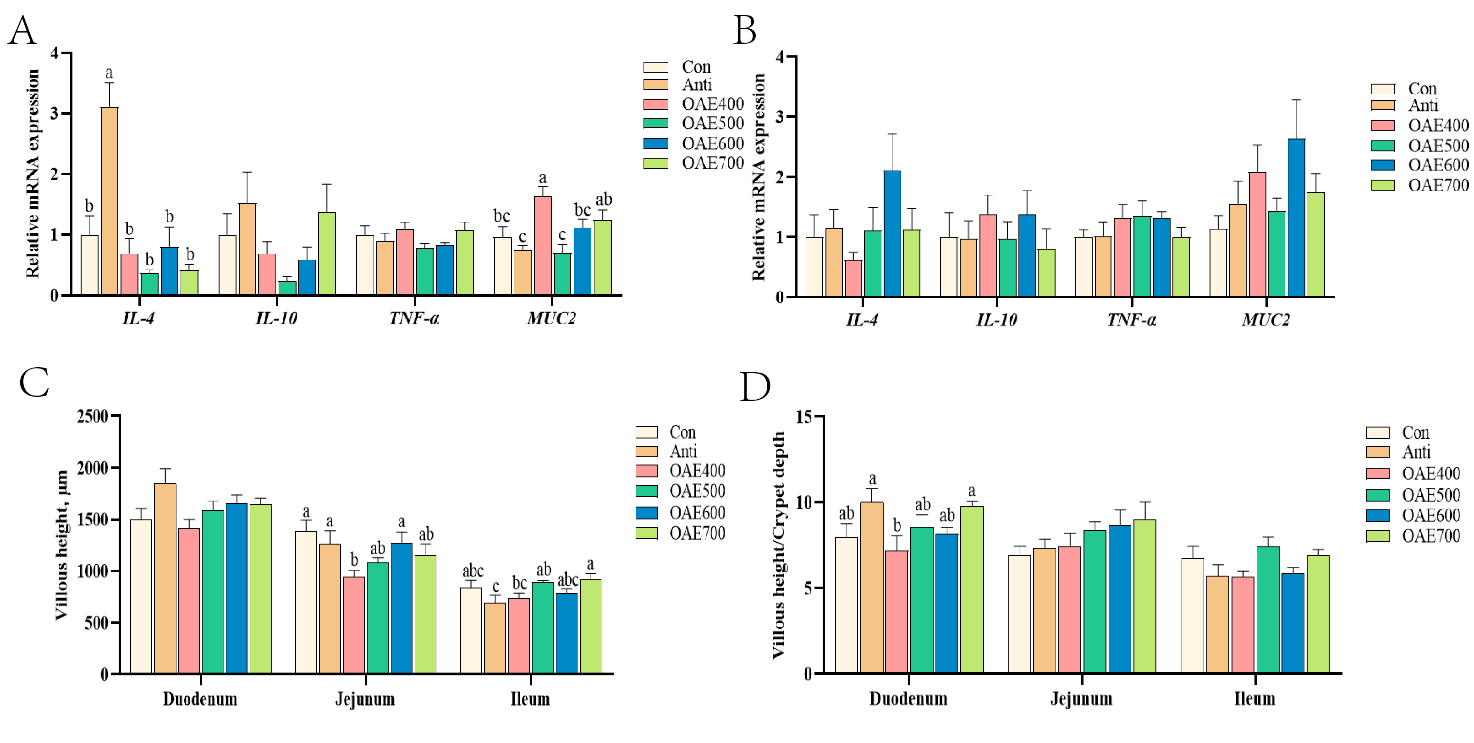


**Fig. S2** OAE affected intestinal morphology and mucosal immunity. The relative mRNA expression of IL-4, IL-10, TNF-α and MUC2 in jejunum (**A**) and [ileum](C:/Program%20Files%20(x86)/Youdao/Dict/8.10.3.0/resultui/html/index.html#/javascript:;) (**B**) at d 21. Intestinal villus height (*C*) and villus height/crypt depth (**D**) at d 42. Data are expressed as means ± standard deviation. ^a−c^Treatments with no common superscripts differ significantly (*P* < 0.05). IL, interleukin; TNF-α, tumor necrosis factor-α; MUC2, mucin 2


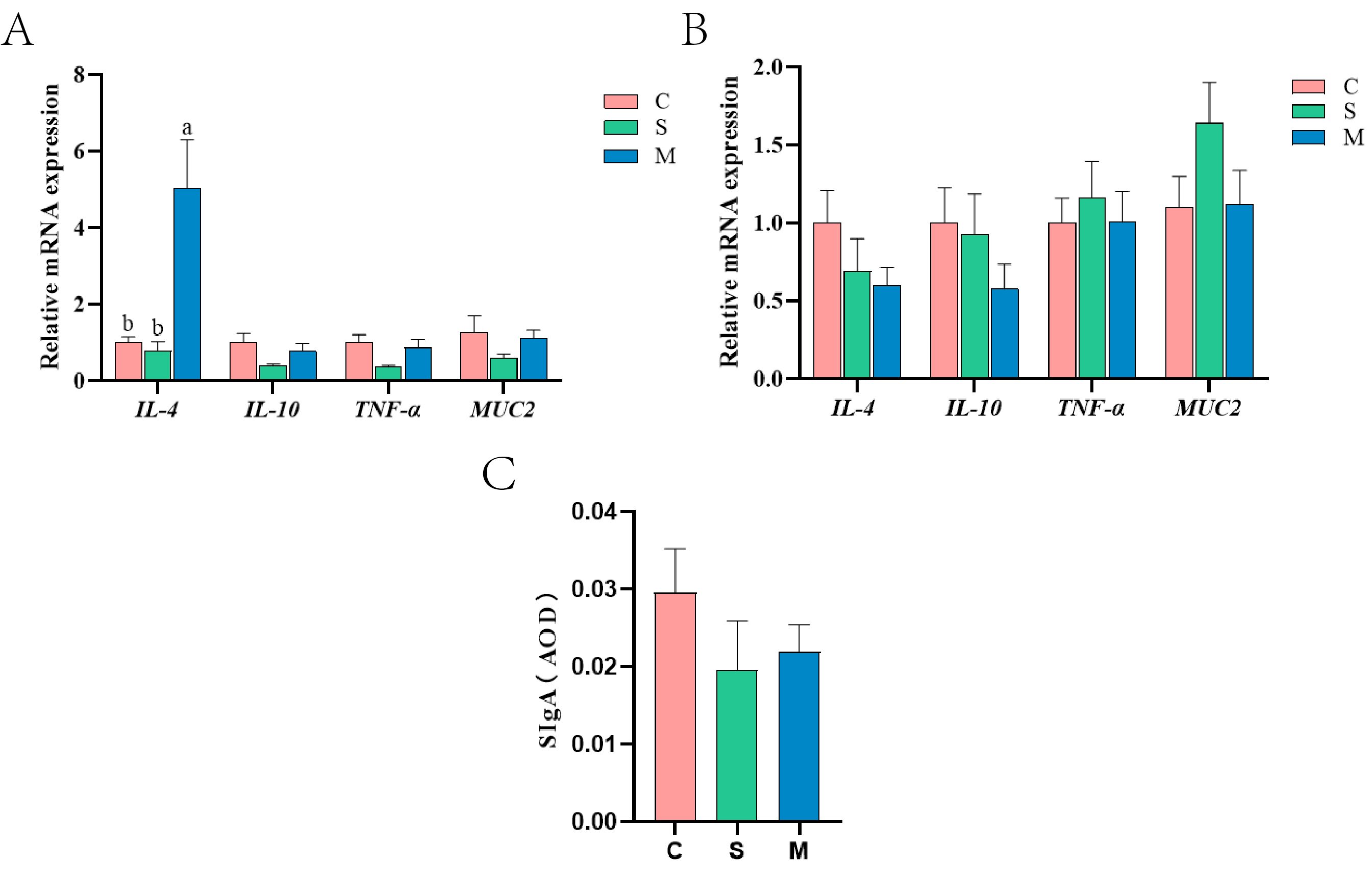


**Fig. S3** [Microorganism](C:/Program%20Files%20(x86)/Youdao/Dict/8.10.3.0/resultui/html/index.html#/javascript:;) and supernatant for intestinal health. The relative mRNA expression of IL-4, IL-10, TNF-α and MUC2 in jejunum (**A**) and ileum (**B**) at d 21. At d 42, the secretion levels of SIgA in jejunum (**C**). Data are expressed as means ± standard deviation. ^a−c^Treatments with no common superscripts differ significantly (*P* < 0.05). IL, interleukin; TNF-α, tumor necrosis factor-α; MUC2, mucin 2; SIgA, Secreted immunoglobulin A
